# Integrated knowledge translation (IKT) in health care: a scoping review

**DOI:** 10.1186/s13012-016-0399-1

**Published:** 2016-03-17

**Authors:** Anna R. Gagliardi, Whitney Berta, Anita Kothari, Jennifer Boyko, Robin Urquhart

**Affiliations:** 1 University Health Network, Toronto, Canada; 2 University of Toronto, Toronto, Canada; 3 University of Western Ontario, London, Canada; 4 Dalhousie University, Halifax, Canada

**Keywords:** Integrated knowledge translation, Decision-making, Health system planning, Scoping review

## Abstract

**Background:**

Integrated knowledge translation (IKT) refers to collaboration between researchers and decision-makers. While advocated as an approach for enhancing the relevance and use of research, IKT is challenging and inconsistently applied. This study sought to inform future IKT practice and research by synthesizing studies that empirically evaluated IKT and identifying knowledge gaps.

**Methods:**

We performed a scoping review. We searched MEDLINE, EMBASE, and the Cochrane Library from 2005 to 2014 for English language studies that evaluated IKT interventions involving researchers and organizational or policy-level decision-makers. Data were extracted on study characteristics, IKT intervention (theory, content, mode, duration, frequency, personnel, participants, timing from initiation, initiator, source of funding, decision-maker involvement), and enablers, barriers, and outcomes reported by studies. We performed content analysis and reported summary statistics.

**Results:**

Thirteen studies were eligible after screening 14,754 titles and reviewing 106 full-text studies. Details about IKT activities were poorly reported, and none were formally based on theory. Studies varied in the number and type of interactions between researchers and decision-makers; meetings were the most common format. All studies reported barriers and facilitators. Studies reported a range of positive and sub-optimal outcomes. Outcomes did not appear to be associated with initiator of the partnership, dedicated funding, partnership maturity, nature of decision-maker involvement, presence or absence of enablers or barriers, or the number of different IKT activities.

**Conclusions:**

The IKT strategies that achieve beneficial outcomes remain unknown. We generated a summary of IKT approaches, enablers, barriers, conditions, and outcomes that can serve as the basis for a future review or for planning ongoing primary research. Future research can contribute to three identified knowledge gaps by examining (1) how different IKT strategies influence outcomes, (2) the relationship between the logic or theory underlying IKT interventions and beneficial outcomes, and (3) when and how decision-makers should be involved in the research process. Future IKT initiatives should more systematically plan and document their design and implementation, and evaluations should report the findings with sufficient detail to reveal how IKT was associated with outcomes.

**Electronic supplementary material:**

The online version of this article (doi:10.1186/s13012-016-0399-1) contains supplementary material, which is available to authorized users.

## Background

It has long been suggested that partnerships between those who produce research and those who use it are likely to enhance the relevance of research and facilitate its use [1, 2]. A variety of terms have been used to label this concept, each are subtly unique, and none are viewed as the over-arching or gold-standard term [3]. In the health sector in Canada and elsewhere, this co-production of knowledge is commonly referred to as integrated knowledge translation (IKT) and defined as an ongoing relationship between researchers and decision-makers (clinicians, managers, policy-makers, etc.) for the purpose of engaging in a mutually beneficial research project or program of research to support decision-making [4]. IKT is viewed as an approach or set of processes that can lead to the generation of knowledge for optimizing health care delivery systems and improving health system performance and associated outcomes [5]. Decision-making research in health care settings shows that complex problems require complex solutions involving input from individuals with different expertise and perspectives and iterative, generative processes to formulate, execute, and evaluate solutions [6]. Collaborative knowledge generation, as promoted through IKT approaches, involves ongoing, dynamic interactions among researchers and decision-makers, and represents an ideal means by which to address complex health care problems [7].

Empirical research in health care settings has demonstrated the concrete benefits of IKT. For example, in-person contact with researchers has been repeatedly cited by decision-makers as the most influential factor determining their use of research evidence [8, 9]. Interviews with participants of nine researcher-decision-maker partnerships funded by the United Kingdom National Health Service revealed that all achieved improved clinical care through a variety of IKT approaches [10]. An exploratory study of a partnership among university administrators and Scottish health authority social workers revealed several impacts including enhanced dialogue among partners about priority health issues, and incorporation of research results into a training curriculum for social workers. As a result, social workers reported the use of research in formal health authority processes and enhanced skill and confidence in using research in their practice [11].

IKT appears to improve the uptake of research into policy and practice through a variety of mechanisms. Collaboration between researchers and decision-makers may reveal differing perspectives, expectations, and values, leading to greater understanding and improved communication, which creates trust and a shared vision that enable more effective and sustained partnership, thereby contributing to the capacity for IKT [12]. On a practical level, decision-makers can inform research questions that are relevant to practice or policy; refine research methods and/or data analysis; interpret findings based on their contextual knowledge; and disseminate or implement findings or products [13]. Decision-makers benefit from interaction with researchers through a broadened reflection on their own activities, enhanced knowledge and skills, information about other pertinent research, and new contacts with other researchers or decision-makers [14]. Researchers benefit as they gain a nuanced understanding of the policy or practice environment, develop and pursue research questions that have real-world applicability, and, through ongoing conversations with decision-makers, interpret results with a deeper understanding of contextual circumstances which, in turn, enhances the usefulness of the research findings.

Despite the emerging evidence of IKT’s positive impact, IKT is not yet widely practiced or well understood. Research directors in Canada reported that researchers tend to use traditional means of conducting and disseminating research rather than IKT approaches [15, 16]. Similarly, a recent survey of health policy experts active in 30 European countries revealed little use of IKT other than isolated instances of embedded researchers in government research institutes or on advisory committees [17]. Lack of engagement in IKT may reflect an inability to overcome the challenges inherent in coordinating complex, protracted initiatives with multiple stakeholders holding different views and pursuing different interests, or it may reflect a lack of incentives for researchers and decision-makers to engage in the more protracted and, hence, costly processes of knowledge co-generation [18, 19]. Reflection on the challenges faced by researcher-decision-maker partners that investigated primary care networks resulted in several recommendations to facilitate IKT including the following: identify partners with pre-established links to ease and expedite interaction; establish clear expectations about role, scope, and contribution to foster trust and avoid role confusion and misconceptions; put in place mechanisms that initiate and support dialogue among partners; and jointly assess progress and implement changes as needed [20]. A case study based on three health service delivery programs found that IKT activities were dynamic and not linear and highly influenced by the complex context within which decisions were being made including social and political norms [21]. When IKT was formally incentivized with considerable funding through national initiatives, the number of interactions and projects increased but the research process was characterized as largely investigator-driven, and there was limited impact on health service delivery and outcomes [22, 23].

Clinicians, researchers, and research funders have emphasized the need to understand how to foster and achieve IKT in the health sector [24–28]. The purpose of this study was to characterize the nature of research in this area, describe IKT strategies that were empirically evaluated, reveal whether sufficient research is available to undertake a systematic review of the effectiveness of various IKT approaches, and also identify knowledge gaps for future IKT research.

## Methods

### Approach

Initially, we had intended on a systematic review; however, based on our preliminary searches which revealed a paucity of studies that have actually evaluated IKT approaches or strategies, we reverted to a scoping review. A scoping review was conducted using approaches promoted by Arksey and O’Malley [29] and Levac et al. [30]. This type of review is used to examine the extent, range, and nature of research activity for a particular topic. A scoping review generates a profile of the existing literature on that topic and identifies gaps, thus serving as a foundation for future reviews or primary research. Such reviews do not attempt to synthesize quantitative findings or assess the quality of the literature. The five-step approach (scoping, searching, screening, data charting, data analysis) was carried out iteratively as the state of the literature on IKT became clearer. We did not assume a theoretical stance or interpretation because that is not customary in a scoping review. Given that this review ultimately did not identify IKT characteristics that lead to beneficial outcomes, consultation with experts to validate or interpret the findings was not carried out [30]. The Preferred Reporting Items for Systematic Reviews and Meta-Analyses (PRISMA) criteria guided the conduct and reporting of the review [31]. Data were publicly available so institutional review board approval was not necessary. A protocol for this review was not registered.

### Scoping the inquiry

To plan for the full-scale scoping review, a preliminary scan of relevant literature was undertaken by searching MEDLINE with the MeSH terms “participatory research” or “interdisciplinary research” or keywords “knowledge exchange” or “integrated knowledge translation.” Search results were first screened by all investigators to begin to understand how IKT was operationalized and then discussed by email and a teleconference. This knowledge was used to establish the research purpose, plan a more comprehensive search strategy, and generate eligibility criteria based on the PICO (population, intervention, comparisons, outcomes) framework. *Populations* refer to researchers and organizational or system-level decision-makers in health care settings including clinician managers, health care managers, and policy-makers involved in academic initiatives (finite or ongoing projects, studies, groups) that used or were based on IKT approaches. Partnerships may have been initiated by researchers or decision-makers where the goal was evidence-informed decision- or policy-making, or they could have a dual purpose—to generate knowledge through empirical research and to resolve practical problems. Non-research partnerships formed solely for quality improvement or to seek input from researchers were not eligible. Studies that focused on front-line providers and clinical decision-making were excluded, as were those focusing on patients or consumers. Although all are legitimate IKT partners, they were considered beyond the boundaries of the current review. The *intervention* of interest was IKT, defined as collaboration between researchers and decision-makers in the research process including establishing the research questions, deciding on the methodology, recruiting and/or collecting the data, interpreting the results, and disseminating the findings [32]. In particular, this review examined the IKT activities that comprised or promoted collaboration, which we refer to collectively as “approaches” but, in the absence of a universally accepted taxonomy, may also be referred to as strategies, mechanisms, methods, activities, or processes. Decision-makers could take part in one or more of these functions but not solely in dissemination or implementation. All studies included in the review explicitly described and evaluated IKT strategies. Study *comparisons* may have evaluated different IKT approaches and associated barriers, enablers and impacts, either alone or in comparison with typical, non-IKT approaches to research, or with other types of approaches for promoting collaborative research. *Outcomes* included but were not limited to knowledge, attitudes, beliefs, partnership formation (shared understanding of issues, common language, etc.), behaviors, and outcomes, while recognizing that one objective of the scoping review was to identify the range of reported impacts.

Eligible study designs included randomized controlled trials, interrupted time series, observational studies (retrospective, prospective, before-after or comparative cohorts), surveys, qualitative research, case studies, or mixed methods research. Studies were not eligible if they

- concluded that IKT was needed without having described and evaluated it
- described the planning or development of an IKT initiative without having evaluated it
- examined issues of authorship among research collaborators
- focused on online communities (i.e., interaction or data collection by social media), translational research (i.e., from wet lab to clinical application), or collaborations between physicians and industry
- described action research, community-based interventions, practice-based quality improvement initiatives (researchers describe conditions in the setting within which they are embedded where the overall goal is quality improvement), practice-based research networks (groups of clinicians or institutions that jointly deliver patient care), or interorganizational networks or quality improvement collaboratives that sought to disseminate knowledge to front-line providers or improve service delivery and outcomes but do not undertake research
- were publications in the form of editorials, opinion articles, protocols, abstracts, proceedings, or conceptual analyses
- if the description of the partnership lacked detail such that it was unclear if decision-makers participated in research activities
- or if research methods used to evaluate the IKT initiative were not provided.

Systematic reviews were not eligible but were used to identify eligible primary studies.

### Search strategy and screening process

A comprehensive literature search was conducted by using several indexed sources. The principal investigator (ARG) and a trained research assistant conducted all searches with guidance from a medical librarian. It has been noted by other researchers that the IKT literature is not consistently indexed [33, 34] so several search strategies were tested by the medical librarian to optimize the retrieval of a few IKT citations known to the investigators (specificity) and, in particular, to increase the likelihood that all relevant studies would be retrieved (sensitivity). The MEDLINE search strategy is shown in Additional file 1 (we used the search shown on line 84). MEDLINE, EMBASE, and the Cochrane Library were searched on 1 February 2015 from 2005 to 2014 inclusive. A 10-year time span was likely to capture most relevant literature since IKT is a relatively new phenomenon in the health care sector. Pairs of investigators screened titles and abstracts according to specified eligibility criteria. Rather than resolving selection differences, all those selected by at least one reviewer were retrieved since ultimate judgment about the inclusion must often be reserved until the full text is examined. If more than one publication described a single study and each presented the same data, the most recent was included.

### Data charting and analysis

A data charting form was developed and piloted by the team to collect information on the country in which the research was conducted, study design, underlying theory used to design the IKT intervention or analyze the findings, the IKT intervention, enablers and barriers, and any reported outcomes. Based on the Workgroup for Intervention Development and Evaluation Research (WIDER) reporting checklist [35], details about the intervention included content (nature and goal of the program and/or IKT partnership), mode of delivery (specific types of IKT activities in which partners were involved), duration and/or frequency (timing of IKT activities), participants (who was involved in specific IKT activities), and personnel (who coordinated or led IKT activities). Time from initiation, the entity that initiated the partnership, and source of funding were also noted. All investigators charted data from eligible studies.

Summary statistics were used to describe the number of studies by country, year of publication, and study design; whether the IKT initiatives employed single or multi-faceted interventions; and whether they were designed based on theory. Relational analysis was used to summarize study findings [36]. With this technique, all data from eligible studies were perused to identify each unique instance of an IKT approach or strategy, enabler, barrier, and outcome. This approach allowed gaps in the IKT literature to be identified. These data were added to the IKT approaches or strategies, enablers, barriers, and outcomes identified in studies referenced in the background of this manuscript and then compiled in a summary of IKT conditions, influencing factors, and outcomes. This approach made clear what was known and not known about IKT interventions. To further understand knowledge gaps, we identified potential associations between the characteristics of IKT strategies, contextual factors, and outcomes by categorizing IKT as used in eligible studies based on type of engagement (conceptualization or planning, recruitment or data collection, interpretation, and dissemination or implementation) [33]; time from initiation; entity that initiated the partnership; source of funding; and the reported barriers, enablers, and outcomes. Outcomes were categorized in relation to study objectives as positive (all reported outcomes were positive or improved) or mixed (some reported outcomes were positive/improved and some were negative/not improved).

## Results

### Search results

**Fig. 1:**
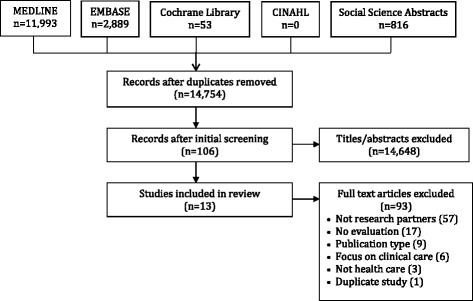
PRISMA diagram of eligible studies

After duplicate titles were removed, the initial search resulted in 14,754 unique articles. Screening of titles and abstracts excluded 14,648 articles, leaving 106 as potentially eligible. Screening of full-text items excluded an additional 93 articles: no partnership (57), no evaluation (17), ineligible publication type (9), focus on clinical quality improvement (6), not health care (3), duplicate study (1), leaving 13 that were eligible for inclusion (Fig. 1). Charted data appears in Additional file 2 [37–49].

### Characteristics of eligible studies

Studies were published between 2005 and 2014. Ten of 13 studies were published in 2013 or 2014 and one each in 2005, 2006, and 2009. Four studies were conducted in Canada, four in the UK, two in the USA, and one each in Lebanon, the Netherlands, and Sweden. Five studies were based on a mixed methods design, five on a case study design, and three on qualitative interviews.

### IKT activities used in partnerships

No studies explicitly mentioned the use of theory upon which the IKT initiative or any of its component strategies were based. Table 1 summarizes the characteristics of IKT partnerships in eligible studies. The content or focus of partnerships varied from specific health topics, for example, implementation of a depression intervention [38] or studying the impact of environment on breast cancer [45], to very broad initiatives that conducted applied health services research to ultimately improve population health [47]. The most common activities or modes of interaction were meetings (i.e., team, working group, committee, board) or presentations (i.e., conferences, workshops). Five of 13 studies reported the duration and/or frequency of specific activities [37–39, 46, 47]. While 8 of 13 studies mentioned the categories of participants involved, 1 of those studies reported the number and/or type of participants involved in specific activities [38]. While all but 4 of 13 studies mentioned modes of interaction [39, 41, 43, 44], authors provided few specific details about when these activities took place and who was involved. For example, in one study, authors reported that team meetings involving an unspecified number of senior managers and medical directors were held periodically over a 3-year period [46]. No studies reported personnel who organized or led the activities.

**Table 1:** Description of IKT initiatives in included studies according to WIDER criteria [35]

Study	Content (program focus)	Mode (IKT approaches/activities)	Duration, frequency, timing	Participants	Personnel
El-Jardali 2014 [38]	Evidence-based health policy-making	Evidence briefs, deliberative dialogues, priority setting, training sessions, rapid response service, web portal	NR	Researchers, policy-makers, other stakeholders from many countries (NR by activity)	NR
Eriksson 2014 [39]	Health promotion	Consultation, meetings, conferences, annual progress reports, joint research, steering group, coordinating committee, working groups	Varied from monthly to annual meetings	Politicians, public clinicians, agency representatives, researchers (NR by activity)	NR
Khodyakov 2014 [40]	Depression	Meetings, working groups, training sessions, web portal	Biweekly meetings, 4 months	Researchers, clinicians, social workers, policy-makers, counselors, clergy (mean 20–25 by event)	NR
Kothari 2014 [37]	Women’s health	Team meetings, priority setting, applying for research funding, joint research, web portal	NR	Researchers, partners, trainees from many countries (NR by activity)	NR
Kislov 2014 [41]	Applied health research on a range of topics	NR	Quarterly meetings, 3 years	NR	NR
Hoeijmakers 2013 [42]	Public health knowledge sharing	Meetings, training sessions, joint research, steering committee, board of governors, public relations	NR	NR	NR
Martin 2013 [43]	Prevention, early detection, self-care, rehabilitation	NR	NR	NR	NR
Murnaghan 2013 [44]	Youth health, prevention of chronic disease	Meetings, planning sessions, presentations; print, web, and media communications	NR	Policy-makers, health authority and agency representatives, researchers (NR per activity)	NR
Rycroft-Malone 2013 [45]	Applied health research on a range of topics	NR	NR	Board, managers, health authorities, committees, researchers (NR per activity)	NR
Soper 2013 [46]	Applied health research on a range of topics	NR	NR	NR	NR
Van Olphen 2009 [47]	Breast cancer	Joint research, meetings, presentations	NR	NR	NR
Patten 2006 [48]	Priority setting practices	Team meetings, joint planning	NR	Clinicians, managers, researchers (NR by activity)	NR
Bowen 2005 [49]	Health promotion	Workshops	Three 2-day yearly events, 5 years	Health authority personnel, researchers (NR by activity)	NR

*NR* not reported

### Enablers and barriers of IKT

Table 2 lists 9 enablers and 15 barriers of IKT reported across studies. All studies reported both barriers and enablers.

**Table 2:** IKT enablers, barriers, and outcomes

Measures	Reported findings	Studies (*n*)
Barriers (9)	Differing needs and priorities among participants	5
	Lack of skill in or understanding of IKT processes	5
	Attitudes about researchers or the value of research	4
	Goals, roles, and expectations not clear	3
	Lack of incentives to participate	3
	Lack of funding or infrastructure for IKT	2
	Little continuity of involvement due to staff turnover, infrequent attendance	2
	Participants are busy with multiple responsibilities	1
	Geographic distant imposes limits on interaction	1
Enablers (15)	Multiple and varied opportunities for interaction	4
	Strong leadership commitment, skill, and experience	3
	Phased approach to develop shared language, achieve early successes	3
	Support from facilitators, champions, and boundary spanners	2
	Clear and agreed upon goals, roles, and expectations	2
	Immersion of researchers in decision-maker setting/co-location	2
	Formalized branding, structures, and processes	2
	Establish partnership early in the research process	1
	Openness of partners to listen, learn, and adapt	1
	Organizational support for decision-makers to meaningfully contribute	1
	Dedicated funding	1
	Shared governance structures	1
	Built on preexisting relationship	1
	Availability of data to inform activities	1
	Periodic external review to assess progress	1
Positive outcomes (12)	Capacity developed by researchers and decision-makers	7
	Decision-makers grew to value research	4
	Developed an appreciation for the collaborative process	3
	Enhanced relevance of the research	3
	Decision-maker involvement sustained through entire process	2
	Enhanced mutual understanding of language, work style, needs, and constraints	2
	Number of collaborative projects undertaken/completed	2
	Influenced policy-making	2
	Influenced service delivery	1
	Increased diversity of involved partners	1
	Strengthened relationships, trust, and goodwill	1
	Emergence of community leaders	1
Mixed outcomes (7)	Decision-maker involvement varied across activities	1
	Failure to overcome differences and bridge boundaries	1
	Collaborations were temporary	1
	Little to no research produced	1
	Research not used in policy-making	1
	Greater emphasis on research publications than stakeholder engagement	1
	Benefits only beginning to emerge	1

### Outcomes of IKT

Table 2 lists the outcomes of IKT that were reported by studies as positive (12) and sub-optimal (7). There was little overlap across studies. For example, the most commonly reported benefit was the development of capacity among researchers and decision-makers for engaging in IKT in 7 studies, and the next most commonly reported benefit was enhanced value for research among decision-makers in 4 studies. Many other benefits and all sub-optimal outcomes were unique to individual studies. Most studies reported measures that reflected the more immediate impact of IKT on partnership formation, for example, mutual understanding of language, work style, needs and constraints, or general views about research or the collaborative process. Fewer studies reported measures that reflected the interim or longer term impact of IKT. For example, 4 studies assessed whether research was used for decision-making.

### Characterization of IKT initiatives

Table 3 characterizes the IKT partnerships, strategies, and conditions in eligible studies. Most partnerships were initiated by governments that provided dedicated resources for the initiative (7 of 13 studies). This did not appear to be associated with successful outcomes. Of the 7 studies with dedicated funding from government, 2 achieved positive, 4 achieved mixed, and 1 achieved sub-optimal or no impact on measures that were reported. All 8 studies that reported timing of the evaluation with respect to time from initiation of the partnership had existed for a minimum of 2 years; thus, partnership maturity did not appear to be associated with outcomes. The number of different types of interaction between researchers and decision-makers did not appear to be associated with outcomes; in the 4 studies that achieved positive impact in all outcomes reported, the number of types of interaction ranged from 1 to 8 (based on data in “Mode” column of Table 1). Many studies did not explicitly report how decision-makers were involved throughout the research trajectory. Among those that provided such details, decision-makers were most often involved in conceptualizing or planning research (10 studies) and in disseminating or implementing the findings (7 studies). One study reported that decision-makers were involved throughout the research process and achieved positive results on all reported outcomes. In contrast, three studies achieved positive results on all reported outcomes when decision-makers were involved in only some aspects of the research process.

**Table 3:** Summary of IKT conditions, influencing factors, and outcomes

Study	Time from initiation (years)	Types of interaction (number)	Initiator/Funding	Decision-maker involvement				Enablers	Barriers	Outcomes^a^
				Conceptualize and plan	Recruit or collect data	Interpret findings	Disseminate or implement			
El-Jardali 2014 [38]	2 to 3	6	WHO Dedicated	+	NR	+	+	+	+	+/−
Eriksson 2014 [39]	NR	8	Government Dedicated	+	+	+	+	+	+	+
Khodyakov 2014 [40]	NR	4	Researcher Research	+	NR	NR	+	+	+	+
Kislov 2014 [41]	2	NR	Government Dedicated	+	NR	NR	+	NR	+	-
Kothari 2014 [37]	2	5	NR Research	+	+	NR	+	+	+	+/−
Hoeijmakers 2013 [42]	2	6	Government Dedicated	+	NR	NR	NR	+	+	+/−
Martin 2013 [43]	2	NR	Government Dedicated	NR	NR	NR	NR	+	+	+/−
Murnaghan 2013 [44]	NR	6	Government Dedicated	+	NR	+	+	+	NR	+
Rycroft-Malone 2013 [45]	3 to 4	NR	Government Dedicated	NR	NR	NR	NR	+	+	+/−
Soper 2013 [46]	NR	NR	Government Dedicated	NR	NR	NR	NR	+	+	+/−
Van Olphen 2009 [47]	NR	3	Researcher Research	+	NR	NR	+	+	+	+/−
Patten 2006 [48]	3	2	Health region NR	+	NR	+	---	+	+	NR
Bowen 2005 [49]	Over 5 years	1	NR Research	+	NR	+	NR	+	+	+

^a^Outcome refers to beneficial or sub-optimal outcomes as reported by studies: in relation to study objectives

+ all reported outcomes were positive or improved, +/− mixed outcomes (reported outcomes positive/improved and negative/not improved), *NR* not reported

### Summary of the IKT conditions, influencing factors, and outcomes

**Fig. 2:**
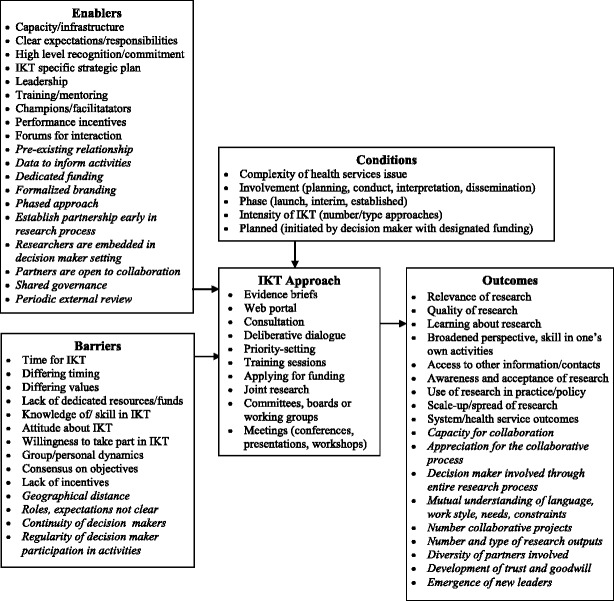
Summary of IKT approaches, influencing factors, and outcomes

Figure 2 shows a summary of IKT approaches, enablers, barriers, conditions, and outcomes that were initially compiled from background literature and expanded with items that emerged from this study. This summary presents the enablers, barriers, and conditions that have been reported to influence the IKT approach and a range of possible outcomes relevant to IKT partnerships. Given the small number of eligible studies, limited detail about IKT, and mixed findings, the relationships between enablers, barriers, contextual conditions, and outcomes remain unclear. As more IKT studies are reported in the literature, the barriers, enablers, and outcomes might be further organized into higher order categories with proposed indicators. In the short term, this framework may be used by others to prospectively plan IKT projects/programs and their evaluation.

## Discussion

This scoping review was conducted to describe the knowledge base underlying IKT, gleaned from studies that described and evaluated IKT strategies, and identify gaps to inform future research. Thirteen studies were eligible. The most common form of interaction was meetings, but they varied in nature, aims, and frequency. All studies reported both barriers and enablers. While most studies achieved one or more positive outcomes, studies reported a wide range of positive and less positive outcomes. Given incomplete and inconsistent reporting of study design, IKT strategies, and outcomes, it was not possible to identify relationships between outcomes and contextual factors related to initiator of the partnership, dedicated funding, partnership maturity, nature of decision-maker involvement, presence or absence of enablers or barriers, or the number of different IKT activities employed in a given initiative. A number of studies assessed partnership formation. Given that the partnerships evaluated were at least 2 years old, it may not be reasonable to evaluate the influence of research on decision-making until more immediate outcomes such as learning about research, awareness and acceptance of research, mutual understanding, development of trust and goodwill, and an appreciation for the collaborative process are established. Another scoping review of stakeholder involvement in rehabilitation research found that stakeholder preparation was needed to understand research and fulfill their role [33]. This took the form of formal and informal training and, in some studies included in that review, decision-makers were paid to participate in the training.

To the best of our knowledge, this study is among the first to attempt to identify the characteristics of IKT strategies and their potential association with outcomes using a rigorous approach. Our scoping review is distinguished from that of Jagosh et al. who published a realist systematic review on the effectiveness of community-based participatory research partnerships that included 276 studies [36]. In participatory, action or community-based research, the intent is to improve the quality of service delivery, health equity, or clinical outcomes where community-identified rather than research-based solutions are emphasized, or researchers function as consultants. We chose to define IKT as partnerships between researchers and organizational or system-level decision-makers including clinician managers, health facility managers, and policy-makers for the purpose of academic research, although those partnerships may have enabled improvements in service delivery or clinical outcomes. IKT decision-maker partners are distinct in that they are specifically selected for their scope of responsibility and, hence, authority to invoke practice or policy change. This scoping review, which goes beyond anecdotal accounts, may serve as a springboard to the conduct of future research that specifically examines researcher-decision-maker partnerships.

Despite suggestions that lack of funding is a deterrent to practicing or achieving IKT [3, 18, 19], in this study, formal IKT partnerships that were specifically initiated and funded by governments did not appear to eliminate barriers or report better outcomes compared with other studies that lacked such infrastructure. This included four studies evaluating Collaborations for Leadership in Applied Health Research and Care (CLAHRC) in the UK [41, 43, 45, 46] and one study evaluating Academic Collaborative Centres (ACC) for Public Health in the Netherlands [42]. Both of these national-level initiatives involved large-scale investment to foster IKT. Since partnerships may develop over time, and additional evaluations may be forthcoming, longitudinal evaluation of these important initiatives is warranted to identify beneficial outcomes. Another way to interpret these findings is that other barriers, enablers, or contextual conditions may be more important than funding to the formation and outcomes of IKT partnerships. Such insight was not afforded by this study because enablers, barriers, and outcomes were variable across studies and not consistently recorded or described. In future research, longitudinal analytic approaches may be useful to evaluate IKT impact and clarify the relationship between IKT approaches and outcomes. A time series design, for example, could be used to systematically track the evolution of partnership formation and better pin-point the activities or strategies that move the partnership from the formation stage into a more functional and active stage.

IKT was poorly and inconsistently described, evaluated, and reported in most studies, making it challenging to identify strong thematic areas. However, three important knowledge gaps were clearly identified. First, some studies evaluated the IKT initiative but did not describe or detail the IKT activities. Future researchers are encouraged, therefore, to capture and report the full extent of IKT activities, including the nature or mode of interactive activities (i.e., brainstorming sessions, data interpretation sessions, passive dissemination through websites), who is involved in which activity, who is leading the activity, and how often activities take place. This cumulative understanding will allow a nuanced typology of different IKT models to emerge. Those who plan and implement and/or evaluate IKT initiatives might employ the WIDER reporting checklist when they design such initiatives or report evaluative findings [34]. The WIDER checklist recommends describing: the intervention (approaches, strategies), mode of delivery (intensity, duration, timing), intervention content (knowledge generated or shared), participants and their role (the characteristics of those sponsoring, delivering, and receiving the intervention), setting, and adherence or fidelity.

The second knowledge gap to emerge was the lack of explicit description of underlying theory or logic upon which IKT approaches and associated activities were selected and/or evaluated. As the WIDER checklist specifies, details are needed about how the IKT intervention was developed, change techniques used in the intervention, and the causal processes targeted by the change techniques to achieve particular outcomes. Therefore, future research could focus on identifying, describing, and testing relevant theory by which to design and/or evaluate IKT initiatives. First, it may be useful to conduct an interpretive synthesis of the findings reported here by analyzing enablers and barriers according to the context and design of IKT initiatives in the included studies. A scoping review is an appropriate starting point to understand the nature of the empirical work in the domain and to determine if a systematic review is warranted. Thus, we deliberately maintained a wide focus rather than targeting certain aspects of the IKT process. Our findings suggest that the empirical work in the area is just emerging, and thus it is premature to embark on a systematic review with a tight focus.

The third knowledge gap pertains to decision-maker involvement. IKT activities most often consisted of meetings between researchers and decision-makers. However, the nature of those meetings and the level of engagement of decision-makers in research-related decisions or research activities were not reported. In some cases, decision-makers were reported as playing a role in disseminating or implementing the results. It was largely not reported if decision-makers took part in any way in the conduct of the research or interpretation of the findings. Given that the nature of decision-maker involvement was largely under-described, we cannot say if the involvement of decision-makers throughout the course of a research initiative, which is the purported ideal [3], actually achieves better outcomes. Other studies of IKT also reported that research remained largely investigator-driven [22, 23], and decision-makers were often not directly involved as integral partners [14]. Future research must examine a range of IKT approaches to identify the ideal timing and manner in which decision-makers must be involved for effective research uptake.

Several issues may limit the interpretation and use of these findings. The relatively small number of eligible studies may have precluded identifying with greater certainty the characteristics and contextual conditions required to foster and achieve IKT. This may, in part, be due to the fact that studies about IKT are difficult to identify. Other researchers have noted that the IKT literature is not consistently indexed in databases of published research [33, 34]. Screening of search results was challenging due to the large number of search results to assess and limited detail in the studies by which to ascertain eligibility. This means that the resulting summary of IKT (Fig. 2) is inclusive of numerous characteristics and conditions that require further evaluation of their association with outcomes. Although we searched standard indexed sources of published medical literature, the search strategy may not have identified all relevant studies. Study retrieval was limited to journals that are indexed in the three databases that were searched. We did not search the grey literature, assuming that most empirical research on IKT interventions would be found in indexed databases. Many studies did not provide a full description of the research methods used or fully describe the research findings for all components of a case study or mixed methods research. Most studies collected qualitative data; however, they were often not complete or sufficiently detailed to extract clear findings with respect to enablers, barriers, and, in particular, outcomes. Given limited detail about IKT activities, it was difficult to chart data; however, we employed a rigorous methodology that complied with standard approaches for scoping reviews [29, 30], and data were charted independently by two investigators for all articles to enhance reliability. In the absence of a universally accepted taxonomy with which to refer to IKT approaches, activities, processes, etc., it was challenging to describe and summarize how IKT was operationalized in included studies. Most studies reported one or more positive outcomes which may represent a bias toward reporting favorable findings; this is further underscored by the small number of eligible studies. Insufficient knowledge emerged from this scoping review to enable a full understanding of the variety of ways to promote IKT partnerships and engage in interactions. Therefore, we are unable to issue clear or justified recommendations in this regard. As more research on IKT emerges, this may become possible at some point in the future. Finally, some scholars [30] have suggested that stakeholders ought to be consulted to validate and extend the interpretation of scoping review findings. Given the variation in results across the 13 articles, we suggest this step is better served in the future when additional research is available.

## Conclusions

This scoping review found that most of the IKT initiatives that were evaluated achieved one or more positive outcomes. However, few studies were eligible, and IKT activities were poorly described, evaluated, and reported. Outcomes did not appear to be associated with initiator of the partnership, dedicated funding, partnership maturity, nature of decision-maker involvement, presence or absence of enablers or barriers, or the number of different IKT activities. Based on these findings, we cannot identify thematic areas across the studies to recommend particular IKT strategies or ideal contextual conditions. However, we generated a summary of the characteristics of IKT that have been examined and identified additional factors that remain to be examined. The findings can serve as the basis for future reviews, and for planning ongoing research that more systematically designs, implements, and evaluates IKT activities, and reports the findings with sufficient detail to reveal how IKT was associated with outcomes. Three important knowledge gaps were identified that lay the foundation for a research agenda in the area of IKT research.

## Availability of supporting data

The data set(s) supporting the results of this article is (are) included within the article and its additional file(s).

## Additional files

Additional file 1: MEDLINE search strategy. (DOCX 17 kb)

Additional file 2: Data extracted from eligible studies. (DOCX 40 kb)
