# Supplementary material for: Integrated knowledge translation (IKT) in health care: a scoping review
Source: Implement Sci. 2016 Mar 17;11:38. doi: 10.1186/s13012-016-0399-1 (PMC4797171; doi:10.1186/s13012-016-0399-1)
Supplement: Supplementary file 1 — MEDLINE search strategy. (DOCX 17 kb) [file 13012_2016_399_MOESM1_ESM.docx]

Additional File 1. MEDLINE search strategy

Note: we used the search shown on line 84 which was the broadest approach

| 1 | exp Research/ |
| --- | --- |
| 2 | Research Design/ |
| 3 | exp Health Services Research/ |
| 4 | exp Biomedical Research/ |
| 5 | exp Qualitative Research/ |
| 6 | Research Support as Topic/ |
| 7 | research.tw,kw. |
| 8 | or/1-7 [Research] |
|  |  |
| 9 | Community-Based Participatory Research/ |
| 10 | Comparative Effectiveness Research/ |
| 11 | Translational Medical Research/ |
| 12 | Knowledge/ |
| 13 | (integrat* adj2 knowledg* adj2 translat*).mp. |
| 14 | (knowledge adj2 translat*).mp. |
| 15 | (knowledge adj2 synthes*).mp. |
| 16 | (knowledge adj2 disseminat*).mp. |
| 17 | (knowledge adj2 exchang*).mp. |
| 18 | Information Dissemination/ |
| 19 | (information adj2 disseminat*).mp. |
| 20 | engaged scholarship.mp. |
| 21 | participatory research.mp. |
| 22 | action research.mp. |
| 23 | or/9-22 [ Integrated Knowledge Translation & related terms] |
|  |  |
| 24 | academic medical centers/ or exp hospitals, teaching/ |
| 25 | "Academies and Institutes"/ |
| 26 | Universities/ |
| 27 | Financing, Government/ |
| 28 | federal government/ or exp united states government agencies/ or government agencies/ or local government/ or state government/ |
| 29 | Government Programs/ |
| 30 | Government Regulation/ |
| 31 | interdisciplinary communication/ |
| 32 | Interinstitutional Relations/ |
| 33 | internationality/ or international cooperation/ |
| 34 | National Health Programs/ |
| 35 | exp "United States Dept. of Health and Human Services"/ |
| 36 | Public-Private Sector Partnerships/ |
| 37 | Drug Industry/ |
| 38 | Laboratories/ |
| 39 | interagenc*.mp. |
| 40 | inter-agenc*.mp. |
| 41 | (academic adj2 government??).mp. |
| 42 | Community-Institutional Relations/ |
| 43 | Public Sector/ |
| 44 | multi-institution*.mp. |
| 45 | multiinstitution*.mp. |
| 46 | multisector?.mp. |
| 47 | multi-sector?.mp. |
| 48 | academia.tw,kw. |
| 49 | research organi#ation?.mp. |
| 50 | Government Agencies/ |
| 51 | (collaborative adj1 centre?).mp. |
| 52 | (collaborative adj1 center?).mp. |
| 53 | or/24-52 [Agencies Involved] |
|  |  |
| 54 | 8 and 23 and 53 [ Option 1: Research + Integrated Knowledge Transfer + Agencies Involved ] |
|  |  |
| 55 | 8 and 53 [ Option 2: Research + Agencies Involved ] |
|  |  |
| 56 | 23 and 53 [ Option 3: IKT + Agencies Involved] |
|  |  |
| 57 | (8 or 23) and 53 [ Option 4: (Research OR IKT) + Agencies Involved ==> Broadest search] |
|  |  |
| 58 | exp program evaluation/ |
| 59 | exp evaluation studies/ |
| 60 | comparative studies/ |
| 61 | evaluat*.mp. |
| 62 | efficacy.mp. |
| 63 | efficacies.mp. |
| 64 | effectiveness.mp. |
| 65 | Health Impact Assessment/ [New MeSH as of 2013] |
| 66 | "Process Assessment (Health Care)"/ |
| 67 | assessment?.tw,kw. |
| 68 | Evaluation Studies as Topic/ |
| 69 | mixed method?.mp. |
| 70 | Validation Studies/ |
| 71 | Validation Studies as Topic/ |
| 72 | or/58-71 [Evaluation & related terms] |
|  |  |
| 73 | 54 and 72 [ Option 1: Research + Integrated Knowledge Transfer + Agencies Involved + Evaluation] |
| 74 | limit 73 to yr="2005 -2014" |
| 75 | limit 74 to english |
| 76 | remove duplicates from 75 [ ===> Option 1: Research + Integrated Knowledge Transfer + Agencies Involved + Evaluation] |
|  |  |
| 77 | 55 and 72 [ Option 2: Research + Agencies Involved + Evaluation] |
| 78 | limit 77 to yr="2005 -2014" |
| 79 | limit 78 to english |
|  |  |
| 80 | 56 and 72 [ Option 3: IKT + Agencies Involved + Evaluation ] |
| 81 | limit 80 to yr="2005 -2014" |
| 82 | limit 81 to english |
| 83 | remove duplicates from 82 [ ===> Option 3: IKT + Agencies Involved + Evaluation ] |
|  |  |
| 84 | 57 and 72 [ Option 4: (Research OR IKT) + Agencies Involved (Broadest) + Evaluation] |
| 85 | limit 84 to yr="2005 -2014" |
| 86 | limit 85 to english |
