# Supplementary material for: Integrated knowledge translation (IKT) in health care: a scoping review
Source: Implement Sci. 2016 Mar 17;11:38. doi: 10.1186/s13012-016-0399-1 (PMC4797171; doi:10.1186/s13012-016-0399-1)
Supplement: Supplementary file 2 — Data extracted from eligible studies. (DOCX 40 kb) [file 13012_2016_399_MOESM2_ESM.docx]

Additional File 2. Data extracted from eligible studies

| Study | Research Design | | | Intervention Design | | Impact |
| --- | --- | --- | --- | --- | --- | --- |
| El-Jardali  2014 [38]  Lebanon | Mixed methods  23 partnership leaders, 17 policy makers/stakeholders and 7 researchers were interviewed; analysis of a report; observation of deliberations two or three years from initiation | | | Content: To enhance evidence-informed health policymaking in ten low- and middle-income countries via partnerships with researchers  Mode: developed evidence briefs, convened deliberative dialogues, held priority-setting exercises and capacity building sessions, offered rapid response services, and developed online clearinghouses  Duration/Frequency: NR  Participants: researchers, policymakers, and other stakeholders from Argentina, Bangladesh, Nigeria, Burkino Faso, Cameroon, Central African Republic, Ethiopia, Uganda, Sudan, and Zambia hosted by NGOs, universities, private research institutions, hospitals, or ministries of health.  Personnel: NR  Theory: NR  Initiated by: international agency (WHO)  Funding source: dedicated | | Facilitators   - Policy-makers’ and stakeholders’ support - International funding support - Strong leadership and political will - Skilled human resources to moderate deliberative dialogues - Location within Ministry of Health brings KTPs closer to policymakers and stakeholders   Barriers   - Lack of skilled human resources to undertake KT activities, including push efforts and facilitating user-pull - Gaps in infrastructure (e.g., lack of functional Internet connection) - Lag in or lack of local research production - Poor quality of local information; difficulty in accessing and finding local evidence - High turnover in top level policymakers in government - Resistance to change and strong political influences - Difficulty in convincing policymakers, stakeholders, and researchers to interact   Outcomes   - Deliberative dialogues were considered useful - Interviewees unable to specify how research evidence was used in policymaking, research production were limited, the impact of implementing evidence into policy had not been assessed - 7 partnerships reported increased awareness of the importance of evidence-informed health policymaking - 8 reported strengthened relationships among policymakers, stakeholders, and researchers - 6 reported their evidence briefs helped inform policymaking at the government level - 6 reported increased demand for information by policymakers - 3 reported enhanced capacity for developing evidence briefs and deliberative dialogues - 1 reported enhanced capacity among policymakers for accessing, assessing, and using research evidence |
| Eriksson  2014 [39]  Sweden | Case study  Reflective dialogues, evaluation meetings, and interviews (numbers NR, years from initiation NR) | | | Content: 3 research, practice, policy partnerships focused on health promotion in alcohol/drug prevention, social inclusion and urban governance, empowering families  Mode: Case 1: consultations, two conferences, annual project leader meetings, annual progress reports on projects and research; Case 2: steering group, coordinating committee, working groups, and annual conferences; Case 3: steering group, joint working groups that met monthly  Duration/Frequency: Varied from monthly meetings to annual conferences.  Participants: NR (politicians, public health professionals, representatives of national agencies, researchers)  Personnel: NR  Theory: NR  Initiated by: government  Funding source: dedicated | | Facilitators   - Contexts that foster a trustful partnership with respectful recognition of each partner’s competence and interests. The researchers need to be trusted by practitioners and politicians. - Highly involved partners. - Politicians were closely involved in two of cases, and their involvement was regarded as critical by the practitioners and researchers as well as politicians themselves. - Dedicating upfront time on joint planning (discussing common goals and producing written agreements). - During data collection and capacity building, it is important to have shared objectives for and dialogues about research.   Outcomes   - Development of culturally appropriate and logistically sound research. Contributing to this development was partner involvement in all facets of research (shaping scope and direction of research, developing research protocols, implementing protocols, and interpreting/disseminating research findings. - Generated capacity to recruit – authors note the development of relationships during the research planning phase was critical to this impact. - Developed capacity and competence of stakeholders. - Contributed to an increase in the quality of outputs and outcomes over time through repeated successful partnering. - Generated new unanticipated projects and activity. |
| Khodyakov  2014 [40]  US | Mixed methods  Observation/minutes from 16 meetings, reflection sheets from 127 meeting participants, 43 survey respondents, interviews with 13 participants and 18 community and academic project leaders (time from initiation NR) | | | Content: implementation of a depression intervention in two community settings via academic-community partnerships  Mode: Mostly meetings, often involving smaller working groups to discuss/accomplish tasks; one service area held two two-day training conference for service providers; online social networking tool to allow interaction between agencies as needed  Duration/Frequency: Biweekly meetings (8 meetings per service area over 4 month period)  Participants: researchers, medical professionals, social workers, counselors, representatives from the local Department of Mental Health, clergy, representations of parks and recreation programs, religious congregations, etc. Mean 20 participants per meeting in one community, mean 25 in the other community  Personnel: NR  Theory: NR  Initiated by: researcher  Funding source: research | | Facilitators   - Leadership structure of the planning process, which consisted of community and academic partners with prior experience working on partnered projects, was essential for improving the capacity of the agencies to work together to develop an intervention plan. - Small group format (e.g., working groups and breakout sessions) of meetings allowed participants to interact with each other in a more meaningful manner, and resulted in the idea of holding training conferences, making it easier to understand how collaboration among a diverse group of agencies may take place.   Barriers   - Meeting attendance - Participant diversity - Lack of clarity on CEP participants’ roles - The process of relationship building was challenged by the fact that some agencies could not send their representatives to all meetings, sent different representatives to different meetings, or stopped their participation in the initiative.   Outcomes   - Project was able to successfully reach the goal of engagement as indicated by (1) the participation of roughly three-fifths of agencies randomized into the study intervention arm, (2) existence of a core group of participants who remained actively engaged throughout the planning process, (3) diversity among the participating agencies many of which do not offer any depression-related services, (4) emergence of community leaders in each service area, (5) and community participants’ positive opinions about group dynamics. - Many community participants reported they were actively engaged because of the participatory features of the project that distinguish it from traditional academic studies. At the same time, this also created confusion among community participants who, especially at the beginning of the process, did not fully understand what the project expected of them or how to collaborate with either potential agencies that do not offer traditional depression care. - Community leadership had a positive impact on group dynamics: it increased meeting interactivity; meeting agendas started to better reflect community needs; and group discussions became focused on issues that were identified as pivotal by agency representatives rather than academic partners. - The goal of collaborative planning was achieved because (1) the toolkits were adapted to the community needs; (2) agencies were trained on intervention components with several individuals performing the roles of trainers; and (3) multiple training conferences on depression care for agency representatives were offered, in which several CEP council members led sessions. - Successful collaboration among diverse agencies required that they understood what was expected of them, were comfortable with the role they chose to perform, and had organizational support to meaningfully contribute to the project. |
| Kislov  2014 [41]  UK | Case study  43 interviews with researchers, clinicians, managers, and executives; 69 hours of meeting observation; and analysis of documents (number NR) at two years from initiation. | | | Content: collaborative partnership between universities and NHS organizations to produce and implement applied health research  Mode: NR  Duration/Frequency: 3 years, quarterly meetings  Participants: NR  Personnel: NR  Theory: NR  Initiated by: government  Funding source: dedicated | | Barriers:   - Research and implementation “strands” were structurally and functionally separated - Researchers and implementers viewed NHS context, and nature, purpose, and aims of the partnership differently - Incentives to participate were different: researchers “hoped to produce high-quality research publications”, while implementers sought to achieve the stated project objectives - Consequently, implementers prioritized service improvement and knowledge translation over research, while researchers prioritized “research implementation” over “doing implementation” - Quarterly research and implementation meetings were ineffective and did not foster “increased interaction, connectivity, and collaboration” perhaps because fundamental aspects like misalignment and competing objectives were never acknowledged or addressed - Even within the implementation “strand”, there were distinct groups that developed their own approaches to implementation and disdained those of other groups, this persisted due to the autonomy afforded the teams   Outcomes   - Effectiveness was impacted by fragmented organizational structure, divergent meanings and identities (misalignment), and a failure to acknowledge these discontinuities and bridge boundaries |
| Kothari  2014 [37]  Canada | Mixed methods  37/75 completed questionnaire; 19 took part in interviews two years from initiation | | | Content: To generate and share knowledge of the links between mental health, substance abuse, gender, and child maltreatment and intimate partner violence, and preventive strategies via an international research network  Mode: team meetings, competitive rounds of seed grant funding (partners including on grants), research projects, Delphi research priority setting process, website  Duration/Frequency: NR  Participants: network members (75) included researchers (41), partners (19), and trainees (15)  Personnel: NR  Theory: NR  Initiated by: NR  Funding source: research | | Facilitators   - Networking was the key perceived benefit, leading to joint writing of papers, working on grants, and speaking at conferences/workshops   Barriers   - Less than half of questionnaire respondents reported a common language/lexicon was being used, or that roles, expectations, criteria for deliverables were explicit - Partners needed more actionable insights to determine how to apply the research   Outcomes   - Knowledge user partner involvement varied across activities, ranging from 11% to 79% participation rates - Partners valued the network at both an individual level and to fulfill their organizations’ mandates - The network enabled partners to readily contact researchers, and partners felt comfortable acting as an intermediary between the network and their own organization. Partners said they used the network as a source for synthesized information. - Most participants used network-generated knowledge in a conceptual way to change or augment their own understanding of violence, resilience, and even data collection and analysis. - Benefits were just emerging |
| Hoeijmakers  2013 [42]  Netherlands | Mixed methods  Interviews with 35 graduate students, 15 practitioners; 5 focus groups with unspecified number of policy makers, researchers, public health professionals; network analysis survey of 34 managers and 69 public health professionals, progress reports from 12 participants and the program lead two years from initiation | | | Content: to improve public health knowledge sharing between researchers, practitioners and policy makers in one region of 19 municipalities  Mode: conduct of joint research, research training opportunities for graduate students, meetings, steering committee, board of governors, public relations  Duration/Frequency: NR  Participants:  Personnel: NR  Theory: NR  Initiated by: government  Funding source: dedicated | | Impact   - Graduate students functioned as boundary spanners between academic and public health settings so cross-domain interactions did not change - Number of collaborative projects and actors increased, however this did not evolve into permanent collaborations at the tactical and operational levels - Research efforts focused on publication rather than implementation so there was little engagement of practitioners and policy makers |
| Martin  2013 [43]  UK | Qualitative  Interviews with 27 leads, deputy leads, executives from government and academic organizations, and coordinators who functioned as boundary spanners two years after initiation | | | Content: Carry out applied health research based on local needs in four themes (prevention, early detection, education and self-management, and rehabilitation) through researcher-research user partnerships from one university and nine agencies  Mode: NR  Duration/Frequency: NR  Participants: NR  Personnel: NR  Theory: NR  Initiated by: government  Funding source: dedicated | | Facilitators   - Formal funding, structures and processes resulted in innovative research and enabled partnerships - Collective sense-making about the ultimate destination and the best way to reach it was achieved through national meetings - External review that identified areas lacking in progress prompted a shift in vision to address implementation and capacity building   Barriers   - Lack of a clear, specific and agreed upon mission/vision - Academic incentives and reporting to the funder incentivized traditional measures of research success (ie. publications) - Most of the funding targeted to research - Given incentives, some academics unwilling to release funds for implementation/capacity-building activities - Government structures/processes and views about the value of research challenged greater involvement of its managers in partnership activities   Outcomes   - Broad range of applied health research projects were developed - Novelty and ingenuity of a programme that made connections across previously isolated research in different chronic diseases - Still, some perceived little change and recommended greater engagement of government staff in research processes - A coherent set of goals were beginning to emerge - Shift in emphasis from relatively traditional applied research towards more novel, implementation-focused activities - Considered alternative measures that were less traditional by which to gauge impact |
| Murnaghan  2013 [44]  Canada | Case study  Case 1 – analysis of 137 documents, 32 interviews, 6 focus groups with 35 participants; Case 2 – 78 documents, 32 interviews, 2 focus groups with 48 participants; Case 3 – 119 documents, 26 interviews, 69 survey respondents, 7 focus groups with 50 participants (time from initiation not reported) | | | Content: To collect and apply data that would improve youth health and chronic disease prevention planning and evaluation via partnerships in three provinces  Mode: Reports, facts sheets, websites, summary fact sheets, newsletters, project summaries, conference proceedings, and media communications were used to share knowledge; meetings, presentations and planning sessions  Duration/Frequency: NR  Participants: Representatives of provincial health/wellness and education government departments, non-governmental organizations, regional health authorities, schools and school districts, universities, and other key stakeholders  Personnel: NR  Theory: NR  Initiated by: government  Funding source: dedicated | | Facilitators:   - Existing system frameworks that identified key processes, people and contextual conditions. - State of readiness - health-related data to inform policy or practice development at local, provincial or national levels - Stakeholders expressed interest in establishing youth health knowledge exchange activities - Multiple opportunities to share information through resources, communications and meetings - Regional and provincial knowledge exchange champions - Leadership and established collaborations between researchers and research users were a crucial basis - Pre-existing national networks provided the initial structure from which to establish new relationships - Success stories were important sources of motivation and learning - Data generated were used to plan, prioritize and evaluate   Outcomes  Stakeholders were helped to interpret and apply evidence including applying surveillance results, assessing priorities, engaging partners and leveraging funding. |
| Rycroft-Malone  2013 [45]  UK | Case study  Case 1: observation of two meetings, interviews with 20 coordinators, leaders, researchers and clinicians; Case 2: observation of one meeting, interviews with 8 coordinators, leaders and researchers; Case 3: observation of one meeting, interviews with 10 coordinators leaders and researchers three and four years from initiation | | | Content: Carry out applied health research through partnership from academic, government and health service settings  Mode: NR  Duration/Frequency: NR  Participants/Personnel: Case 1 – one university and four NHS trusts; three research programs focusing on heart disease, depression, and diabetes; a director, deputy director and manager provide leadership; board provides oversight; Case 2 – two universities, seven NHS trusts, five primary care trusts and five other organizations including a charity; research focuses on self-management and on implementation; an executive board and a strategic board with representatives of all involved agencies provide leadership; Case 3 – one university and seven NHS trusts; research themes include prevention, early detection, self-management and rehabilitation, and implementation for older people, health education, reducing emergency admissions, improving primary care services and improving secondary care services; a director, associate director, manager, board and executive committee provide leadership  Theory: NR  Initiated by: government  Funding source: dedicated | | - There were disagreements within cases about how to interpret their mission and approach - Approaches were influenced by participants’ interpretation of whether implementation was a collaborative activity - physical co-location influenced views about implementation - Implementation was operationalized in four ways: quality improvement, translating national guidelines into practice, sharing evidence throughout the organization, and generating and using local evidence - Integrated and shared governance structures influenced the potential for implementation by enabling collaboration - Dedicated resources operationalized as boundary spanners or linking agents enabled collaboration between health services and academia, however some participating clinicians informally assumed this role - Resources also enabled activities from formal events like meetings to creating informal space that enabled idea sharing - Formal branding of the partnerships also prompted knowledge exchange - Engagement and prioritization were influenced by historical tensions between service and academia, different professional agendas and competing priorities, and the view held by health services that researchers were to help them rather than seeing themselves as integral partners - There was a lack of shared values, language and understanding |
| Soper  2013 [46]  UK | Case study  Case 1 - interviews with 12, one-day workshop with 25; Case 2 – interviews with 17 and one-day workshop with 14; participants included service users, policy makers, managers, local authorities, researchers, and clinicians (time from initiation not reported) | | | Content: Case 1 - partnerships between a foundation, university and health and social care providers to improve community mental health; Case 2 – partnership between 2 universities and all health service providers in one region to improve health outcomes for patients and the public  Mode: NR  Duration/Frequency: NR  Participants: NR  Personnel: NR  Theory: NR  Initiated by: government  Funding source: dedicated | | Facilitators   - Evolved out of a previous collaboration - Many relationships pre-existed - Exploited local capacity, ie. systematic review centre - Case 1 - only one clinical focus, ie. mental health - Case 2 – acquired outside research funding - Willingness to listen, adapt and learn among all participants - Quick wins seen by managers helpful to further engagement - Adequate funding for patient/public involvement   Barriers/Challenges   - Changes in government policy, focus on short-term priorities given budget constraints, staff turnover in positions of authority, disappearance of organizations - Lack of clarity of goals and focus, distinction between research and implementation was unclear - Unclear about how to achieve goals - Challenging to engage GPs, middle managers, government commissioners, local authorities - Competing requirements of different service providers and patient groups - Difficult for some researchers to balance need for speedy and relevant answers with research rigour   Outcomes   - The approach used, understanding of how to deliver mission, and level of decision-maker engagement evolved over time - Measure of research success evolved from completion of research studies to include demonstration of engagement - Researchers grew to value engaging with clinicians - Learning each other’s language, style of working, incentives and constraints - Strengthened relationships, trust and goodwill - Capacity developed in both academic and health service communities; fertile environment to conduct and use research - Service providers better appreciated health services research - Range of successful projects undertaken with clear impact on service delivery - Many projects initiated by, and involved patients - Acquired £17 million in external research funding, published high-quality research |
| Van Olphen 2009 [47]  US | Mixed methods  Rating of partnership approach, interviews and a focus group with 12 community members, researchers and others (time from initiation not reported) | | | Content: Partnership of researchers from three academic organizations, personnel from three public health departments, community based organizations and breast cancer survivors to study the environmental impact on breast cancer etiology  Mode: decision-makers functioned as co-investigators (details NR) and participated in dissemination (educational materials, publications, presentations, town hall meetings)  Duration/Frequency: NR  Participants: NR  Personnel: NR  Theory: NR  Initiated by: researchers  Funding source: research | | Facilitators   - Annual town hall meetings enabled the community to provide input, and findings to be shared with the community - Researchers gained understanding of community concerns; conveyed research in ways that were more meaningful to the lay public - Research questions and data interpretation were enriched - Interaction strengthened relationships, and created trust and understanding, which made the research more responsive to community needs   Barriers   - Budget and timeline limited full community engagement - One research study was funded to answer specific questions with a particular research design so not possible for community to provide input - Lack of skill or knowledge to participate in research in a meaningful way - Partners with differing priorities and needs - Mistrust of researchers, research perceived as not benefiting the community - Communicating across stakeholder groups to convey value of research, stakeholders widely dispersed   Outcomes   - 9/12 (75%) returned quantitative rating form - At least half of the respondents highly rated the following:   - Target users included in the research process   - Efforts were made to build trust between researchers, users   - Users were provided with opportunity to learn about research methods   - Research questions were jointly developed   - User knowledge/experience used to conceptualize, design and implement the research - Community members brought diverse concerns to researchers, participated in the research process, and shared findings with the community |
| Patten  2006 [48]  Canada | Qualitative  A focus group with 8 senior managers, interviews with 8 senior managers at 2 years from initiation, and 17 interviews with senior managers and clinicians at 3 years from initiation | | | Content: partnership of researchers with leaders from a regional health authority to develop and implement priority setting practices  Mode: team meetings, joint planning  Duration/Frequency: periodic meetings over 3 years  Participants: senior managers and medical directors (numbers not reported)  Personnel: NR  Theory: NR  Initiated by: regional health authority  Funding source: NR | | Facilitators   - Real pressures on decision makers to address a large budget deficit provided a “strong internal impetus” to develop and implement the PMBA model - “Win-win”: dual management of health economists’ consultant/researcher role “lended credibility to the research project” and afforded professional development opportunities for CHR participants - Deep knowledge of context gained by researchers was important to the success (customization and contextual appropriateness) of the project/model. - Project proceeded naturally through phases of “action, research and change interventions” and this was considered important in terms of acknowledging the challenges associated with change amongst the CHR participants including overcoming natural human resistance/reluctance to change - Internal commitment and strong proponents of the process - External objectivity described as valuable in building a fair and rigorous process - Strengthening partnerships was referenced as being important to building mutual understanding both within the CHR, and between CHR and researchers   Barriers   - Need for change to be identified by management - Fluidity of researcher role as educator, process facilitator, health economist, content expert, management consultant, health researcher - Recognizing change as an incremental process - Capacity building through intense collaboration for knowledge and skill transfer - Merging theory and practice, recognizing the theory-practice divide |
| Bowen  2005 [49]  Canada | | Qualitative  Interviews with 101 regional health authority CEOs, managers and clinicians, policy makers and researchers throughout the course of the five-year project | Content: partnership between researchers and two regional health authorities to generate research knowledge of relevance to planning within that would ultimately improve population health in those regions  Mode: workshops  Participants: researchers and regional health authority representatives (number NR)  Duration/Frequency: 3 2-day workshops were held annually each of the five years  Personnel: NR  Theory: NR  Initiated by: NR  Funding source: research | | Facilitators   - To build mutual trust and authentic and respectful peer relationships, participants identified both structured (e.g., meetings in which objectives were set and achieved) and informal opportunities (e.g., team dinners) to interact - Understanding of context, where it was particularly important for research team members to achieve a true sense of the resource limitations of the community partners and the constraints they faced in terms of time - Development of a shared language and culture to encourage participation in team discussion - Recognition that building trust within the team, developing a shared culture, and identifying common priorities requires time - Strong, trustful relationships and the building of networks were key to collaborative knowledge creation   Barriers   - Trust identified as an initial barrier, particularly as it pertained to the merit of initiative; much of this initial lack of trust appeared to relate to the novelty of the relationship and a lack of mutual understanding amongst researchers and RHA members - Lack of research understanding and use among community partners - Greatest challenge associated with project success was identified as “moving from individual capacity-building to influencing how research is used for planning within the regions”   Outcomes   - Three kinds of learning were experienced by region team members over the course of the project: factual learning (e.g., research concepts, findings of specific research projects); how to locate and access needed information; and a change in how research was viewed and their relationship to it - Researchers identified important learnings as they related to: the importance of team-building with their community partners; an appreciation for the expertise and context-relevant knowledge possessed by the regional team members, including the resources constraints they faced; real barriers regarding research understanding and use; a genuine understanding of the import of knowledge translation - Three levels of project impact were identified: individual learning of team participants related to conceptual use of research rather than application; changes in how regions made decisions; changes to networks and committees at the provincial level whereby meetings were conducted differently as a consequence of the project and expectations have changed (been raised) regarding how collaborative and productive (planning) meetings should be. | |
